# Ferroelectric Domain Modulation with Tip-Poling Engineering in BiFeO_3_ Films

**DOI:** 10.3390/mi15111352

**Published:** 2024-11-05

**Authors:** Xiaojun Qiao, Yuxuan Wu, Wenping Geng, Xiujian Chou

**Affiliations:** Science and Technology on Electronic Test and Measurement Laboratory, North University of China, Taiyuan 030051, China; wuyuxuan1224@163.com (Y.W.); chouxiujian@nuc.edu.cn (X.C.)

**Keywords:** ferroelectric films, BiFeO_3_, domain reversal, charged domain wall

## Abstract

BiFeO_3_ (BFO) films with ferroelectricity are the most promising candidates regarding the next generation of storage devices and sensors. The comprehensive understanding of ferroelectric switchable properties is challenging and critical to robust domain wall nanoelectronics. Herein, the domain dynamic was explored in detail under external bias conditions using scanning probe microscopy, which is meaningful for the understanding of domain dynamics and the foundation of ferroelectric devices. The results show that domain reversal occurred under external electric fields with sufficient energy excitation, combined with the existence of a charged domain wall. These findings extend the domain dynamic and current paths in ferroelectric films and shed light on the potential applications for ferroelectric devices.

## 1. Introduction

Lead-free ferroelectric BiFeO_3_ materials have attracted enormous attention owing to domain modulation with varying polarization [1,2,3]. Compared with other ferroelectric materials, BFO exhibits a significant domain-switching process and various topologies [2,3]. Recently, it has been reported that the domain modulation and charged domain walls could be exploited in nanoelectronic devices, including high-density ferroelectric random access memory, logic devices, and photovoltaic sensors [4,5,6,7], the basic principles of which are nanoscale domain modulation and charge gathering near domain walls. Numerous researchers have explored the domain dynamic modulation and enhancement of domain wall conductivity, including strain engineering [8], electrical bias, and forcing conditions [9,10], as a promising aspect. Among them, electrical bias is an efficient method owing to the adjustable operation. Regarding the electrical modulation of the ferroelectric domain, numerous studies have been carried out on ferroelectric materials, including PZT [11,12], LiNbO_3_ [13,14], and BiFeO_3_ films [15,16,17]. All the results show that the electrical bias is a feasible way to nanoscale domain engineering. In addition, the reversed domain could last for a period and be immune to the external environment range [18]. Since the nanoscale domain is a critical component for nanoelectrons, it is urgent to explore the domain dynamic under external electrical bias, especially the processes of domain nucleation, growth, and merging with the neighbor region. Until now, studies have mainly focused on the operation of the device suffering from fatigue and temperature effect [19]. Few studies have conducted a compact investigation of domain dynamics within the nanoscale, which is quite meaningful for the understanding of ferroelectric nature and for paving the way for next-generation devices.

In this work, domain reversal was explored under tip-poling engineering with various poling conditions. The typical ferroelectric property was confirmed, revealing robust domain adjustability. Also, the threshold value of domain switching was determined through a series of poling processes, which is beneficial for device consideration. Except for the domain reversal, it was interesting to observe the domain wall conductivity near the domain wall, which is tunable when compared with the domain itself. All these results indicate that domain engineering with tip poling could be an effective way for domain modulation in BFO films, which is meaningful for the design of storage devices and domain wall-based electronic devices.

## 2. Materials and Methods

Ferroelectric BiFeO_3_ films were prepared using the pulse laser deposition method, which could be found in previous studies [20]. Briefly, the BFO films were grown on the SrTiO_3_ substrates using the PLD method with a KrF 248 nm laser with an energy density of approximately 2 J/cm^2^ and a repetition rate of 6 Hz. The distance between the target and substrate is about 50 mm.

The topography and domain wall conductivity of films were measured using AFM (MFP-3D, Asylum Research, Santa Barbara, CA, USA) and ORCA™ (ORCA) with the software of AR 15.0, which facilitates conductive AFM imaging using the MFP-3D AFM [21]. To explore the domain dynamic, various electrical biases were exploited on the top surface, and the domain reversal process was characterized by a piezoelectric module using PFM mode, during which a conductive tip with a force constant of 2.5 N m^−1^ was used. The typical butterfly-like curve was obtained through switching spectrum piezoresponse force microscopy (SS-PFM) under room temperature conditions [22,23].

## 3. Results

**Figure 1:**
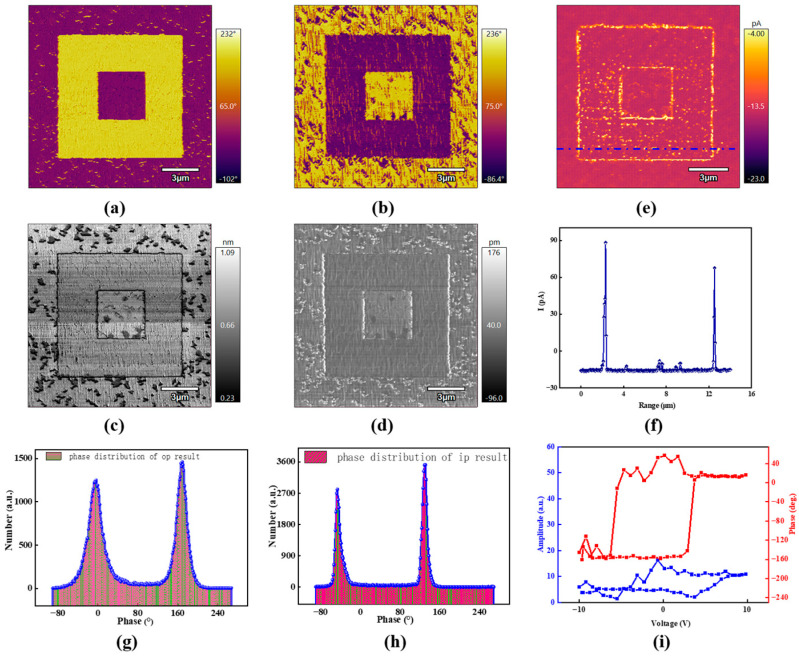
The domain reversal using the predefined pattern under a tip bias of 15 V: the out-of-plane (**a**) and in-plane (**b**) phases, and the corresponding amplitudes in (**c**,**d**). The phase distribution of domain variants regarding the in-plane and out-of-plane phases is shown in the inset of (**c**,**d**); the domain wall conductivity (**e**) and section-line profile (**f**) in the BFO films, (**g**,**h**) refer to the distribution of domain in both the out-of-plane and in-plane direction. (**i**) Characterization of the coercive field.

Figure 1 shows the domain-switching property under external electrical bias. The electrical bias is applied to the Pt-coated tip with opposite polarity. These poling regions exhibit obvious domain reversal under the tip bias of 15 V. According to the reported domain variants; the domain is pointed towards the upward direction with a 180° domain wall interface [24]. Normally, the domain reverses to the opposite direction under the tip bias, which is typical in ferroelectric materials. Regarding the specific poling parts, the domain turns in the downward direction with a positive bias, while the other region keeps the original state with a negative electrical bias. The domain-switching process could be confirmed via phase distribution, as shown in Figure 1g, by the phase difference between the upward and downward domains. Except for out-of-plane domain reversal, BFO films exhibit interesting in-plane domains according to previous studies that used angle-modulated scanning probe microscopy. The in-plane domain also exhibits obvious switching properties, as shown in Figure 1b; the corresponding phase distribution is shown in Figure 1h, revealing the controllable modulation prospect with the tip-poling engineering. The domain wall location was obvious near the domain pattern with the opposite direction shown in Figure 1c,d. It can be noted that the phase distribution of the domain variants was consistently focused on two regions, as shown in Figure 1g,h. In addition, enhanced conductivity with an amplitude of ~60 pA was observed near the domain wall under a sample bias of 2.5 V, as shown in Figure 1e,f. Regarding the mechanism of domain wall conductivity, several reports have been conducted to confirm that the conductivity arises from the charged carrier compensation with different poling conditions [25,26,27].

**Figure 2:**
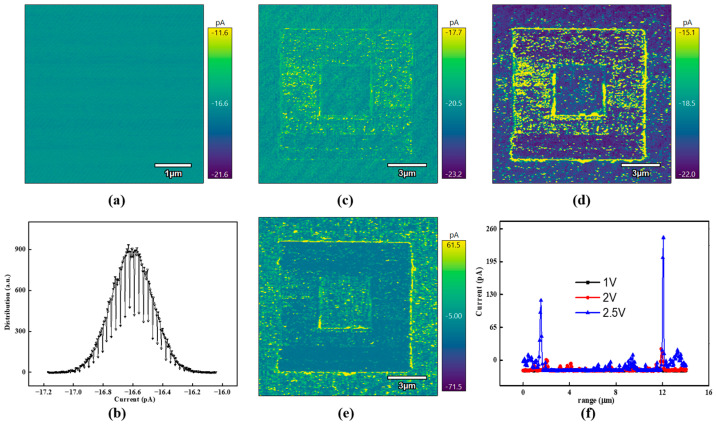
The conductivity of BFO films in the as-grown state (**a**,**b**); corresponding conductivity under various electrical biases 1 V, 2 V, and 2.5 V, respectively (**c**–**e**); the domain wall conductivity profile (**f**) according to the current results of c-AFM.

Figure 2 shows the domain wall conductivity using the ORCA™ mode. During the current measurement, the contact force is quite critical owing to the protection of the contact interface. The conductive probe was grounded with the substrate being biased. To ensure sufficient contact during the current measurement, a probe with a relatively high elastic constant was used in the current measurement. The original domain exhibits lower conductivity with an amplitude of ~16 pA, as shown in Figure 2a,b, where the corresponding domain variant and domain wall have been conducted. According to the previous studies [17,28], the domain wall conductivity could be modulated under external bias. In this poling region, the conductivity of the domain wall was explored with various biases, showing the enhanced current level near the domain wall with an amplitude of several hundred pA, as seen in Figure 2c–f, which is meaningful for nanoscale electronic devices.

**Figure 3:**
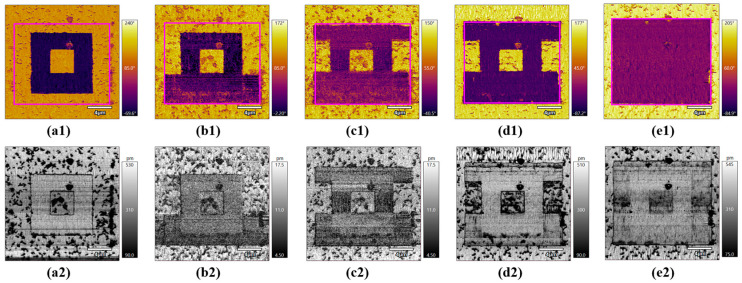
The domain dynamic process in the previous poled region with electrical biases of −5 V, −5.5 V, −6 V, −6.5 V, and −7 V, (**a1**–**e1**) phase and (**a2**–**e2**) amplitude imagines respectively; the poling region was as below: first, the poling was biased in the center region with dimensions of 8 μm × 8 μm, which is similar to Figure 1. After that, a series bias of −5 V, −5.5 V, −6 V, −6.5 V, and −7 V was applied with the dimensions of 14 μm × 14 μm shown in pink remarks in (**a1**–**e1**); note that the scanning area is 19 μm × 19 μm to analyze the domain switching.

Through the various domain dynamics with an internal threshold value of 2 V, we can observe that the domain reversal process occurred when the applied bias exceeded the threshold value. Further, to fix the detailed domain dynamic under an intermate state, the detailed domain dynamic was explored based on the previous domain pattern (the pink area in Figure 3). Various electrical biases from −5 V to −7 V were applied to the tip, and the corresponding phase contrast was obtained simultaneously, as shown in Figure 3(a1–e1), combined with the corresponding amplitude images shown in Figure 3(a2–e2). The obvious domain nucleates and expands immediately when an external bias of −5.5 V is applied and gradually merges with the neighbor region. The out-of-plane domain exhibits a switching trend under the tip bias [29,30]. With a larger bias of −7 V, the poling area changes into one domain orientation, indicating that sufficient poling energy promotes the domain-switching process.

**Figure 4:**
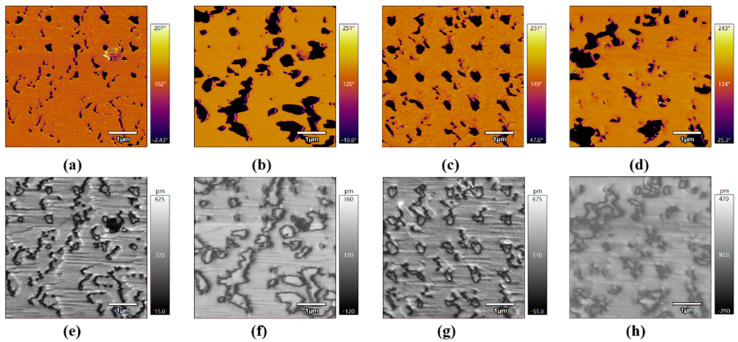
The domain dynamic using point-step poling engineering: out-of-plane (**a**,**c**) and in-plane (**b**,**d**) phases with the corresponding amplitude shown in (**e**–**h**). For (**a**,**b**), the electrical bias ranges from −1 V to −13 V, with a 0.1 s poling increment; for (**c**,**d**); the electrical bias is constant at −14 V with a 0.2 s poling increment.

In addition to the poling process with a predefined pattern, another poling mode was conducted with step-by step poling engineering. Figure 4 shows the corresponding domain reversal under various point-poling conditions. With lower bias shown in Figure 4a,b, domain switching cannot occur; for the higher voltage of −7 V, the out-of-plane domain reversal process could be seen, which is correlated with the nucleation-limited switching mode. Further enhancement of poling bias promotes the corresponding in-plane switching shown in Figure 4a,b. To confirm the various dynamics, another poling process was conducted with a tip bias of −14 V and a poling time increase of 0.2 s, as shown in Figure 4c,d. The domain reversal with arrays was clearly observed in both the out-of-plane and the in-plane domain, which is consistent with the predefined poling pattern, indicating that the energy favored-domain switching in the BFO films is closely related to the poling amplitude and constant time [31,32,33].

## 4. Discussion

Domain switching is a critical phenomenon regarding memory and nanoelectronics devices. It is interesting to notice the domain reversal under the tip-poling engineering using piezoresponse force microscopy. It can be noted that the domain-switching process does not occur until enough poling is applied to the crystal system, which is correlated to the coercive fields and consistent with the previous domain dynamic and macroscopic *P*-*E* curves [29]. Upon exceeding the threshold energy, both the out-of-plane and in-plane domain variants reverse along the direction of the applied electrical field, corresponding to the various angles of domain switching.

The most accepted fact is that under an external electrical bias, domains are prone to reversing, and the positive and negative charge centers shift under stress and lattice distortion. When the condition of minimizing the system’s free energy is met, spontaneous polarization microregions are formed. The stability of switching domains needs to be explored through experiments, and different crystal materials have different stable polarization states in the ferroelectric phase. The stable state of ferroelectric domains is closely related to the shielding state, including the electrical and mechanical boundary conditions, as well as the environmental atmosphere. In addition, it is also related to stress matching, defect vacancies, and film thickness during the material preparation process.

Ferroelectric films usually do not have the conditions for the formation of charged domain walls, which means that charged domain walls could hardly be observed in an as-grown state [32]. In the experiment, polarization reversal was achieved by loading an external electric field, leading to discontinuous polarization near domain walls and the gathering of charges [28,32]. During the polarization voltage loading process, the polarization parameters were adjusted or the stress state changed to induce the stable domain switching, leading to the corresponding conductivity near domain walls. In other words, the formation of domain wall conductivity is closely related to stable domain states and complete shielding characteristics. The energy characteristics at the interface of the ferroelectric domain are jointly determined by the internal charge carriers and energy barriers of the system. Different polarization orientations form domain walls with different energy states. Generally speaking, the response of domain walls at interfaces with different orientations is completely opposite. When the domain orientation is head-to-head or tail-to-tail, injected charges, and conductive carriers migrate and aggregate under the Coulomb force of the domain walls. These types of domain walls are usually referred to as charged domain walls and are closely related to the domain stability and screening effect [30,33].

## 5. Conclusions

In this work, the domain dynamic and domain wall conductivity in BFO films were investigated with tip-poling engineering using piezoresponse force microscopy. The results show that the domain dynamic process obeys the traditional nucleated-limited model, the domain reversal process occurred with the energy-favorable condition, and it merged with the neighbor region. The enhanced domain wall conductivity was obtained, which is consistent with the domain-switching process and closely related to the polarization discontinuous state along the domain wall. The conductivity was quite stable under the test conditions. All these results indicate that electrically adjustable domain reversal is effective in modulating the domain dynamic at the nanoscale. This understanding of domain dynamics expands the ferroelectric-based phenomenon and is meaningful for advanced electrical devices.

## Data Availability

Data availability statements are available upon reasonable requirements.
